# Nanomaterials in Biomedicine 2022

**DOI:** 10.3390/ijms24109026

**Published:** 2023-05-19

**Authors:** Daniel Arcos

**Affiliations:** 1 Departamento de Química en Ciencias Farmacéuticas, Facultad de Farmacia, Universidad Complutense de Madrid, Instituto de Investigación Sanitaria Hospital 12 de Octubre i+12, Plaza Ramón y Cajal s/n, 28040 Madrid, Spain; arcosd@ucm.es; 2 CIBER de Bioingeniería, Biomateriales y Nanomedicina, CIBER-BBN, ISCIII, 28040 Madrid, Spain

Nanomaterials in biomedicine are materials designed at a scale of 1–100 nanometers that make it possible to diagnose, treat and prevent diseases using tools and knowledge of the human body at the molecular scale. Since its beginnings in the 1980s, the strong development of nanotechnology has driven extraordinary advances in fields such as computing, biology and construction, but arguably it has been the advances in medicine that have aroused the greatest interest of society at a global level. Properly designed and synthesized nanomaterials have opened up an enormous range of possibilities for diagnosing, treating and preventing diseases and injuries; relieving pain; and preserving and improving human health.

There are currently approximately 100 nanomedicines on the market and close to 600 that are in clinical trials or at other stages of development [1]. Among those available, more than half are cancer treatments (53%), followed by treatments for infectious diseases (14%), which provides a clear picture of what science and 21st century societies see as the main threats in the field of health. However, in the year 2022, the frontier of knowledge in the field of nanomedicine has also reached regenerative therapies for bone, neural tissue, etc., immunomodulatory strategies, the design of nutraceuticals, advances in drug delivery strategies and the development of new and more effective diagnostic agents, among many other applications. This Special Issue, “Nanomaterials in Biomedicine 2022”, contains eight research papers and a review of some of the most up-to-date aspects of this discipline.

An interesting aspect of nanomaterials used in biomedicine is their ability to modulate drug release kinetics. The article by Nazarkina et al. [2] proposes an interesting strategy based on the incorporation of activated carbon into electrospun polycaprolactone fibers. These fibers can act as a retention barrier in the release of sirolimus from vascular stents, thus ensuring the long-term release of this drug and avoiding vascular restenosis. The results of these authors show that the electrospun fibers containing activated carbon have a biocompatibility similar to that of PCL fibers, demonstrating their capacity to be used as a retention layer that allows delayed drug release over time.

One of the main objectives in the design of nanomedicines is to reduce the adverse effects associated with the systemic administration of antitumor drugs. The functionalization of nanoparticles with targeting agents or the design of stimuli-responsive systems that prevent the early release of the antitumor drug have been widely explored lines of research in this field. However, Zhou et al. [3] propose in their article a different and interesting prophylactic strategy involving the use of rapamycin perfluorocarbon nanoparticles to mitigate the acute toxic effects of cisplatin on the kidney. Using fluorescence microscopic imaging and fluorine magnetic resonance imaging/spectroscopy, the authors show that these nanoparticles are able to reach and be retained in kidneys injured by cisplatin administration. The result is that renal structure and function are preserved 48 h after the administration of the antitumoral drug, offering a new mechanism-based prophylactic therapy for cisplatin-induced acute kidney injury.

The search for new and more effective diagnostic agents is one of the pivotal research fields for the development of new nanomaterials for biomedical applications. In this sense, surface-enhanced Raman spectroscopy (SERS) has proven to be an excellent diagnostic tool thanks to its rapidity and ultra-sensitivity, which allows increasing the Raman spectroscopy signal by several orders of magnitude of the analytes of interest when they are absorbed on functional substrates. The article by Feng et al. [4] demonstrates the effectiveness of faceted Co_3_O_4_ microcrystals for this purpose, depending on the dominantly exposed facets, thus providing new insights into the facet-dependent SERS for semiconductor materials. In the same line of improving the diagnostic quality by SERS, Dallari et al. [5] describe the synthesis and evaluation of a new Raman reporter (RR) obtained from low-cost precursors and requiring three steps of 18 h duration, thus overcoming the limitations of the current reported procedures. By conjugating this RR to gold nanoparticles of high anisotropy through their SH groups, constructs with the ability to act as SERS probes, with high colloidal stability and detection limits below 60 pM are obtained. These nanodevices can be functionalized to present bio-tagging capabilities, opening the possibility of their use for the parallel detection of more analytes for multiplexed encoding and detection.

The incorporation of new synthetic strategies based on more sustainable and ecofriendly procedures is one of the most interesting aspects in the development of new nanomaterials. In their article, Galić et al. [6] propose the preparation of selenium nanoparticles to be evaluated as a potential nutraceutical. For this purpose, they use olive pomace, a valuable form of olive oil production waste rich in antioxidant polyphenols. The authors demonstrate that functionalization with OPE reduces the particle size of Se, improving bioaccessibility in the gastrointestinal environment, although it affects the biocompatibility of the particles depending on the cell line with which it is evaluated.

Graphene oxide has attracted great interest in various fields of science and nanotechnology due to its remarkable electrical, mechanical, chemical, and thermal properties. Therefore, numerous investigations in the field of biomedicine have been carried out to take advantage of the properties of graphene oxide (GO) as well as its reduced derivatives (rGO), arousing great interest in its potential use in neural tissue engineering. In the article by Feito et al. [7], the aspect of the immune response to GO and rGO is further explored by analyzing the specific response of T helper (Th) lymphocytes involved in the adaptive immune response. This paper points out the possible benefits of reducing graphene oxide to improve its biocompatibility and increase its potential for the preparation of scaffolds in tissue engineering.

Finally, this Special Issue includes two articles and a review that address the preparation of bioactive mesoporous glasses (MBG) and their application in bone tissue regenerative therapies as well as in dental resins. The review article [8] comprehensively reviews the advances in the preparation of MBG nanoparticles. These nanomaterials consist of small spherical particles that exhibit chemical properties and porous structures that stimulate bone tissue regeneration. The article discusses the different alternatives that these nanoparticles offer as drug and therapeutic ions carrier systems. In this way, the advances achieved for the treatment of bone pathologies, such as osteoporosis, bone cancer, infection, etc., which are frequently associated with bone tissue loss, are reviewed and discussed.

The article by Jiménez-Holguín et al. [9] describes the preparation of ZnO-substituted MBGs, carrying out the in vivo evaluation of these grafts in a sheep model. The osteoinductive effects of the Zn^2+^ cation had been widely published with in vitro assays on osteoprogenitor cells. However, the Zn^2+^ cation also inhibits the formation of the apatite-like phase on the surface of these nanometer-scale engineered bioceramics, which is necessary for bone tissue regeneration. This article sheds light on the consequences of incorporating Zn^2+^ into MBGs in a biomechanically relevant in vivo model.

Finally, Marovic et al. [10] show how the incorporation of Cu doped MBG nanospheres in resins with applications in dental restoration. The bactericidal properties of copper ions in these types of bioceramics are well known. In this article, the authors show how a combination of Cu-MBG nanoparticles, silica, and inert Ba-glass micro fillers seems to have the best perspective for future clinical use.
